# Multimodal prior knowledge determines false memory formation

**DOI:** 10.1037/xge0001852

**Published:** 2025-11-13

**Authors:** Marco A. Petilli, Francesca M. Rodio, Daniele Gatti, Marco Marelli, Luca Rinaldi

**Affiliations:** 1 Department of Psychology, University of Milano-Bicocca, Milano, Italy; 2 Institute for Advanced Studies, IUSS, Pavia, Italy; 3 Department of Brain and Behavioral Sciences, University of Pavia, Pavia, Italy; 4 NeuroMI, Milan Center for Neuroscience, Milano, Italy; 5 Cognitive Psychology Unit, IRCCS Mondino Foundation, Pavia, Italy

**Keywords:** False memories, multimodal prior knowledge, vector-based models, vision-based similarity, language-based similarity

## Abstract

Memory formation is a complex phenomenon shaped by various experiential traces, yet their exact contributions remain unclear. This study investigates the generation of false memories leveraging different data-driven computational models to independently quantify language-based and vision-based experiential knowledge, as extracted from large-scale databases consisting of 639 billion words and 15 million images, respectively. We then tested the effects of these knowledge sources in two false-memory experiments, one employing images and the other words as stimuli. Our findings unveil both modality-independent and modality-dependent processes in the formation of memory traces. Indeed, we observed a contribution of both prior visual and linguistic knowledge regardless of the types of stimuli to be memorised. However, the extent of this contribution differed as a function of the modality tested: visual prior knowledge is more influential in image-based tasks, while linguistic prior knowledge dominates in word-based tasks. This dual and modality-dependent contribution underscores the adaptive nature of memory processes, revealing the dynamic integration of diverse experiential traces in false memory formation.

Memory is a rich tapestry that is woven by the threads of our experiences. In the absence of sensory deprivation, these experiences encompass images, sounds, touches, smells and flavours, as well as words. These different experiential sources are supposed to dynamically affect the content and the organisational structure of human memory, shaping, in turn, our understanding of the world we live in. Yet, despite its multifaceted nature, research has often focused on isolating single experiential traces, either sensory or linguistic, to understand memory formation. This has led to fragmented accounts, overlooking the dynamic interplay between different types of experiential traces.

The debate has long raged between proponents of grounded cognition, who argue that semantic knowledge in memory is rooted in sensorimotor experiences (Barsalou, 1999, 2008; Fischer, 2012), and advocates of linguistic relativity, who maintain that language would play a crucial role in shaping conceptual and even perceptual processes (Boroditsky, 2011; Kay & Kempton, 1984; Lucy, 1997). These opposing views have been complemented by more integrated perspectives, such as the dual-coding theory (e.g., Paivio,1971; Symbol interdependency, Louwerse, 2011; Andrews et al., 2009, 2014), which emphasises that human memory relies on two functionally independent (although interacting) classes of mental representations: verbal representations and mental images, thus suggesting a complementary contribution of visual and linguistic traces in memory formation.

Despite attempts to reconcile extreme perspectives, recent studies still often emphasise the relevance of one type of experiential trace over the other. For instance, studies have shown that perceptual properties of objects affect human performance even in purely linguistic tasks, where perceptual information is neither solicited nor required (Glenberg & Kaschak, 2002; Lachmair et al., 2011; Petilli et al., 2021). Conversely, linguistic information has been demonstrated to exert a rapid and pervasive influence on perceptual tasks, including visual search, discrimination and recognition (Lupyan, 2008; Lupyan et al., 2020; Lupyan & Ward, 2013; Samaha et al., 2018; Winawer et al., 2007). However, the intricate interplay between multiple experiential traces remains mainly underexplored.

Memory plays a fundamental role in organising concepts, being a crucial cognitive system in determining how we encode, store and retrieve information. Importantly, this system is far from fully reliable, but it is susceptible to false memories and distortions (Schacter, 1999; Vecchi & Gatti, 2020). Indeed, when we recall events or information, human memory engages in an active reconstruction process (Bartlett, 1932; Sulin & Dooling, 1974). This reconstructive process is not random but affected by how consolidated knowledge is interconnected in our memory. Thus, false memories can provide valuable insight into how concepts are organised in human memory. To illustrate this, imagine recalling a visit to a sports equipment store from the last week. In the store, various types of sports equipment were offered, including rugby balls, tennis rackets and volleyball nets. Yet, when you retrieve this event one week later, you may tend to fill in missing details from memory based on existing knowledge and schemas. For example, you may erroneously remember novel objects related to the exposed ones, such as soccer or tennis balls, due to the association of these objects with prior knowledge about sports equipment stored in long-term memory. This example illustrates how false memories may be taken as a window into the structure of human memory itself.

However, false memory is a complex and multifaceted phenomenon, spanning from surface-level distortions (e.g., Hupbach et al., 2007) to deeper false recall and recognition involving words (e.g., Roediger & McDermott, 1995) and even events (e.g., Loftus & Palmer, 1974; Mazzoni & Memon, 2003). Consistent with this, previous studies have discussed the existence of various types of false memories (e.g., Mazzoni, 2002). The relationship between two of the most widely used paradigms (i.e., the DRM and the Loftus’ misinformation paradigm) has been examined by several studies, typically revealing mixed results with weak or inconsistent correlations (e.g., Calvillo et al., 2017; Ost et al., 2013; Zhu et al., 2013). This may partly stem from methodological (rather than theoretical) limitations – for example, the low reliability of the misinformation paradigm, which uses few items, compared to the DRM task, which allows for tens or even hundreds of trials. Given this theoretical and experimental complexity, in the present study, we decided to employ the Deese-Roediger-McDermott (DRM) task (Deese, 1959; Roediger & McDermott, 1995) due to its extremely high reliability, replicability and flexibility. Notably, the DRM is probably the most widely used task to investigate false memories (e.g., Gallo, 2010; Coane et al., 2021). In this task, participants are typically presented first with a list of words that must be memorised (words to be studied, e.g., *hill, valley, climb, summit*, etc.). These words are associatively/semantically related to a non-presented word, named critical lure (e.g., *mountain*). After a brief distracting task, participants are then asked to perform a recognition task, whereby they are presented with memorised words and with the critical lure. During this recognition phase, participants often falsely recognise the lure as part of the memorised list, demonstrating the powerful role of semantic similarity between words in false memory generation (Gallo, 2010).

In the standard DRM approach, associative/semantic relationships between words are mainly established through word association norms and account for a large portion of the variance in false memories (Deese, 1959; Roediger and McDermott, 1995; Roediger et al., 2001 PBR), thus providing a solid demonstration that associative/semantic processes are the primary determinants of false recognition in the DRM task. However, the use of word association norms warrants consideration of several limitations related to the method itself, being it bound to time, geographical and practical constraints; and, most critically, introduces issues from an epistemological standpoint, as it is limited in addressing the deeper question of what source of semantic knowledge originally causes false memories. As indicated by De Deyne et al. (2021), human responses in word associations draw on diverse experiential sources (including language, visual imagery, or the recollection of affective states; Deyne et al., 2021). As a result, the multimodal nature of the semantic information captured by these associations limits their ability to provide insights about which specific components of semantics underlie classic effects in the DRM task.

To overcome these limitations, here we integrated the DRM paradigm with computational models trained on large text corpora and image datasets, offering a powerful method to independently operationalise and evaluate the role played by prior language- and vision-based semantic knowledge – namely, pre-existing knowledge about concepts derived from previous linguistic and visual experience that the brain, or in this case its computational proxy, leverages to process incoming sensory input. Evaluating the effects of computationally derived – rather than human-based – semantic associations on false memory is pivotal in this study, as it allows us to dissect semantic effects typically observed in the DRM task into their foundational independent experiential sources – here visual and linguistic – thus tackling the nature of semantic knowledge that in this task is responsible for false memories.

In recent research, these experiential priors have already been shown to predict memory recognition within their respective – linguistic or visual – modalities: linguistic similarity between words, as induced from text-based distributional semantic models (DSM), was found to predict performance in a word-based DRM task (Gatti et al., 2022), with higher similarity between new and studied words leading to increased false recognition. In a similar vein, it has been demonstrated that also visual similarity between images, as induced by convolutional neural networks (CNNs) for vision tasks, predicts false memories in an image-based DRM task (Děchtěrenko et al., 2021; Rodio et al., 2024), with higher similarity between new and studied images leading to increased false recognition.

Our approach builds on these findings and takes a step further by integrating these computational approaches to examine how both visual and linguistic knowledge predict memory recognition across different stimulus modalities. Specifically, we assessed the simultaneous contribution of these different sources to false memory formation in word- and image-based DRM tasks. This is crucial as it enables a direct comparison of the relative effects of visual and linguistic prior knowledge both within and across their respective stimulus formats, thereby highlighting both modality-specific and multimodal effects as a function of the stimulus format.

We extracted proxies for image-based prior knowledge from a large-scale database containing around 15 million images and proxies for language-based prior knowledge from natural language corpora consisting of 639 billion words. We then used these proxies to construct two ad-hoc variants of the DRM false memory task, employing an automated, data-driven tool for generating memory lists; and, finally, involved human participants in two versions of the DRM tasks, one with images (Experiment 1) and one with words (Experiment 2) as items. Importantly, to eliminate any differences between the two tasks, we designed them to use identical stimuli: images in Experiment 1 and corresponding labels in Experiment 2. Our findings reveal that memory traces are indeed multimodal, with both linguistic and visual prior knowledge shaping our memories. However, the influence of these traces reflects a modality-dependent reliance, in that visual prior knowledge dominates in image-based tasks (Experiment 1), while linguistic prior knowledge is more prominent in word-based tasks (Experiment 2). These findings thus provide a more comprehensive understanding of memory formation, unveiling the dynamic interplay between different experiential traces.

## Computational framework

### Vision-based experiential prior knowledge

**Figure 1:**
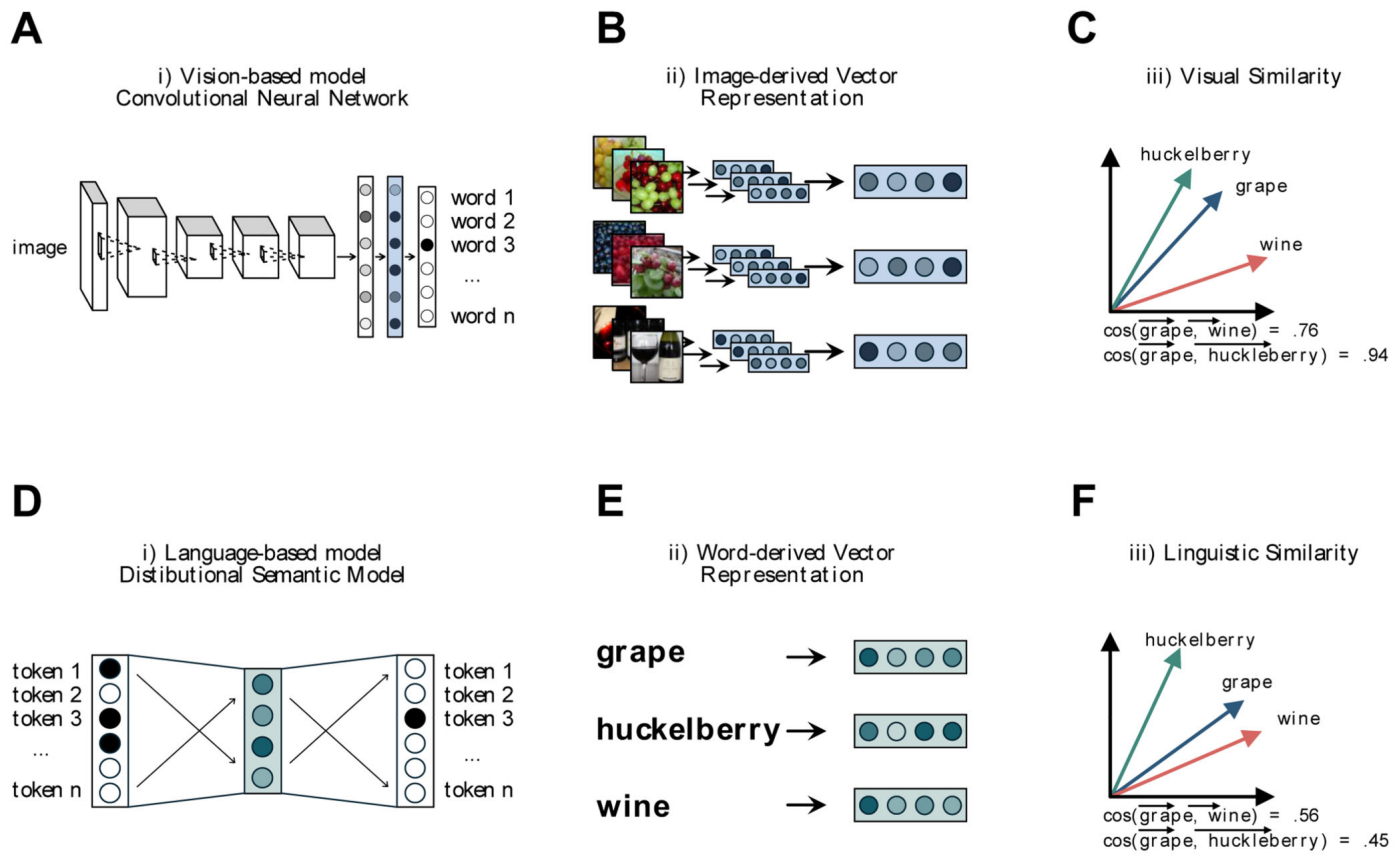
A) Schematic illustration of the convolutional neural network (CNN) applied on images. B) Computation of the visual vector representations for the prototype, derived by averaging the vectors of individual image exemplars within a category. C) Graphical illustration of the estimation of cosine similarity between visual-based representations. D) Schematic illustration of the distributional semantic model (DSM) applied to a large linguistic corpus documenting natural language use. E) Extraction of the linguistic vector representations from the DSM. F) A graphical illustration of the estimation of cosine similarity between language-based representations.

Vision-based vector representations were obtained via a state-of-the-art pre-trained computer vision model for image classification (Krizhevsky et al., 2012). These models, known as convolutional neural networks (CNNs), learn to recognise objects depicted in input images through extensive training with a large set of labelled images. At their core, CNNs consist of a complex, multilayer structure composed of numerous interconnected neurons. The activation of neurons in a layer can be represented as a high-dimensional numerical vector, encoding the visual features available in the input image (Figure 1A). In other words, once an image is provided as input, the CNN breaks it down into a set of numerical values (also known as vector representation or embedding), which represent its visual features – like colour, shapes, patterns, and textures. Each image vector can be represented as a point in a multi-dimensional space of visual features, with each numerical value representing a coordinate. This setup allows us to compute how visually similar two images are by measuring the distance between their points in the space (with images that are visually similar - i.e., with a similar combination of visual features - ending up being mapped to nearby points in the space). In mathematical terms, *vision-based similarity* between two images is computed using a similarity index based on the cosine of the angle formed by their corresponding vectors (Bullinaria & Levy, 2007).

Besides deriving vector representations for individual image exemplars, through these models, it is also possible to extract vision-based vectors for visual prototypes, built on accumulated perceptual knowledge and serving as proxies for visual representations of concepts (Figure 1B) (Petilli, & Günther, 2024). These vectors can be thought of as “aggregated visual experience with the word referents, abstracted from the idiosyncrasies of individual exemplars” (Günther et al., 2023). In mathematical terms, these vector prototypes can be obtained by taking the centroid vector of their respective category, i.e. averaging the vectors of individual image exemplars within that category (e.g., vectors from various images of *dog*) (Anderson et al., 2017; Battleday et al., 2020; Gualdoni et al., 2023; Günther et al., 2023; see also Posner, 1970 for the prototype-distance theoretical conceptualisation). Through this procedure, the visual prototype of a category captures the common visual features shared across multiple image exemplars of the category, such as the overall shape and fur patterns of a dog, while filtering out unique or uncommon features specific to individual images, such as a dog with an atypical ear shape or wearing a sweater. Within the context of this study, prototypical vectors are more suitable than vectors for image exemplars as they provide a visual representation for the referents of words and thus can also be used analogously in linguistic tasks (see, for example, Petilli et al., 2021, in which these vector prototypes were used to predict lexical priming effects; see also Gualdoni et al., 2023, in which vector prototypes were used to predict naming variation) (Figure 1C).

The specific procedure we used to obtain vector prototypes is well illustrated in a recent study by Günther et al. (2023). Following the same approach, we first sampled up to 200 different images with the same labels from ImageNet (Deng et al., 2009) – a large-scale database containing around 15 million labelled images adopting the hierarchical category structure of WordNet (Fellbaum, 1998). Each of these images was then provided as input into the CNN, and we extracted the vector representations induced by the model. Finally, the vector representations within each category were averaged to obtain the prototypical vector representations.

In this study, we used as CNN the pre-trained network VGG-F (Chatfield et al., 2014) available in the MatConvNet library (Vedaldi & Lenc, 2015) in MATLAB. This model consists of 5 convolutional layers followed by 3 fully connected layers. Following previous works (Günther, Petilli, & Marelli, 2020; Günther, Petilli, Vergallito, et al., 2020; Petilli et al., 2021), we considered the activation vector induced by the penultimate fully connected layer consisting of 4096 dimensions. Notably, the validity and psychological plausibility of the vector representations for images and visual prototypes obtained through this approach have been empirically demonstrated in various studies in the cognitive domain (Günther et al., 2023; Peterson et al., 2016, 2018; Petilli et al., 2021; Zhang et al., 2018; for a review see, Petilli & Günther, 2024).

### Language-based experiential priors

Language-based vector representations were obtained via distributional semantics (Landauer & Dumais, 1997; Lenci, 2008; Turney & Pantel, 2010). Distributional semantic models (DSMs) are based on the distributional hypothesis, according to which words with similar meanings tend to appear in similar linguistic contexts (Harris, 1954). Following this principle, distributional semantic models represent the meaning of a word based on its distribution across many language corpora. As a result, words with similar meanings are represented by similar distributional vectors.

Similarly to vector spaces derived from visual models, distributional vectors of words can also be characterised as points within a space, and the similarity between words is represented by the distance between the corresponding points (Figure 1F) (e.g., vectors for words like *wine* and *grape* will occupy nearby points in this space, while *wine* and *huckleberry* would be more distant from each other). Hence, estimating the similarity between the distributional vectors of two words provides a quantitative estimate of the similarity between their meanings as derived from a proxy of prior language experience (what we refer to as *language-based similarity*). Over the years, these vector representations have proven to be effective predictors of human performance in a large variety of psychological tasks (e.g., Baroni et al., 2014; Jones et al., 2006; see Gatti et al., 2022, 2023a, 2023b, for studies in which DSM vector representations predict false memories in a DRM task with words as stimuli; for a review see Mandera et al., 2017), and there is substantial theoretical support for their plausibility as models of human semantic representations (see Günther et al., 2019; Jones, Willits, et al., 2015).

To quantify the role of linguistic experience in this study, we leveraged modern prediction-based DSMs (Mikolov et al., 2013) using the Italian pre-trained vectors from fastText (https://fasttext.cc/; Bojanowski et al., 2017; Grave et al., 2018; Joulin et al., 2016, for a review see: Bonandrini & Gatti, 2024) (Figure 1E). These recent models are based on a neural network architecture designed to predict a target word based on the context of the surrounding words (or vice versa). Specifically, this model was trained on Common Crawl (around 630 billion words) and Wikipedia (around 9 billion words) using the Continuous Bag of Words method with 300 dimensions and a co-occurrence window of 5 words.

Importantly, FastText vectors have been shown to achieve state-of-the-art performance in various semantic tasks (e.g., word similarity, analogy tasks) (Bojanowski et al., 2017). Finally, from a practical standpoint, FastText provides vector representations in a multitude of languages, including Italian, making it a suitable model for our study involving Italian speakers (see https://fasttext.cc/docs/en/crawl-vectors.html).

### DRM Lists Construction

Two parallel versions of the DRM task were created, one using images (Experiment 1) and the other using the words associated with those images (Experiment 2). The selection of the lists of stimuli on the basis of the described models proceeded concurrently. This was done to ensure that each word in the DRM-word variant had a corresponding image in the DRM-image variant.

Images, along with their corresponding English word labels, were obtained from ImageNet (Deng et al., 2009). As a preliminary step, we excluded ImageNet categories for which the label included multiple words or hyphens. We considered all categories comprising at least 100 images, i.e. an adequate number of images to construct reliable visual prototypes (Günther et al., 2023). When a label was associated with multiple categories in ImageNet, we only kept the category with the largest number of images. The dataset was then filtered by excluding infrequent English image tags with Zipf Flickr Frequency (US) lower than 2 (Petilli et al., 2024). Flickr frequency is a hybrid word frequency measure that approximates familiarity with both the images of objects and the terms used to label them. By excluding items with low Flickr frequency, we minimise the risk of including image and word stimuli that are not familiar to participants, thereby reducing the potential introduction of noise or confounding variables into our experiments (e.g., Diana & Reder, 2006).

A second step involved translating the word labels of the remaining ImageNet categories into Italian. For this purpose, we used both DeepL (https://www.deepl.com/) and Google Translator (https://translate.google.com/). When the two translations differed, we selected the one deemed most representative of the specific image category. If neither translation was deemed suitable, the category was excluded. Next, we further filtered the dataset by excluding Italian image tags with a Zipf Flickr Frequency (IT) lower than 2 (Petilli et al., 2024).

Next, we proceeded to select an image representative of each Italian word label. First, we excluded all images with a width-to-height ratio different than 4:3 (i.e., the most common ratio in ImageNet) and a resolution lower than 224 x 299 pixels. All remaining images were resized to a standard resolution of 224 x 299 pixels^1^. This was done to standardise the images and ensure that technical differences, such as size, width-to-height ratio, or resolution, did not influence memory performance. Images were then visually inspected in descending order of image typicality (as computed in Günther et al., 2023) until an image met the following requirements: good graphic quality (no pixelated images), no readable text (such as logos, phrases, or overlaid characters, to avoid memorisation of lexical stimuli in the image task), and category representativeness (to minimise the risk of potential effects of typicality on memorability; Kramer et al., 2023). Categories for which no image met the above criteria were excluded.

After this selection process, we obtained a dataset comprising 1,687 items from distinct ImageNet categories, each associated with a representative image and an Italian label. The mean Zipf Flickr frequency IT for the selected items was 3.6 ± 0.86 standard deviations, while the mean Zipf Flickr frequency US – relative to the items in their original English form – was 4.2 ± 0.85 standard deviations. This indicates that, overall, the final set of items consisted of medium-frequency words in the image dataset – words moderately used to refer to the corresponding visual referents (with the lowest frequencies deliberately excluded during stimulus selection). The list construction procedure resulted in the selection of items belonging to heterogeneous WordNet categories. Most of the items, approximately 41%, represented *artefacts* other than structures – (e.g., chairs, razors, and submarines). Around 15% of the items belonged to the category *animals* and covered a wide range of species from terrestrial and aquatic environments (e.g., falcons, octopuses). About 11% of the items consisted of *structures*, including buildings and architectural spaces (e.g., chapel, tower). Around 8% belonged to the category *person*, including human occupations or societal roles (e.g., fireman, cheerleader). Roughly 7% consisted of *plant and plant-part* items (e.g., seeds, rose). Approximately 7% of the items included *food*, ranging from basic ingredients to complex dishes (e.g., beans, pudding). Finally, around 11% of the items belonged to diverse, less represented categories (e.g., alcohol, natural depression, shape) that did not fit into any of the preceding ones (e.g., liquor, canyon, cylinder).

**Figure 2:**
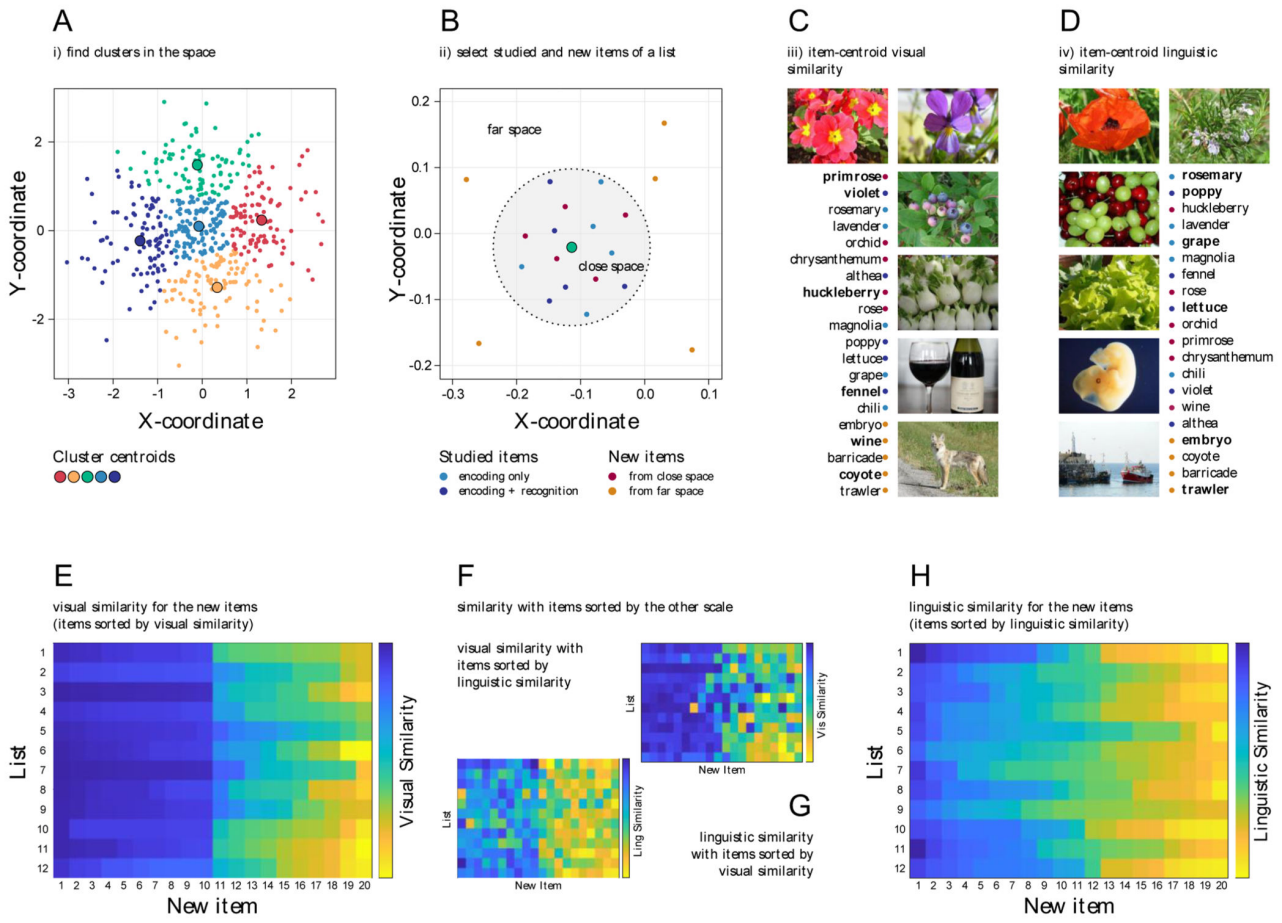
The top panels report a schematic illustration of the DRM list construction. A) The vector space is partitioned into clusters based on their similarity, with one cluster per list. B) The space around each cluster is divided into close and far space. From the close space, the studied items and a set of new items are selected. The remaining new items are selected from the far space. C) Example of items from one list sorted according to their similarity to the centroid of the studied items in the vector space of visual prototypes (i.e., visual similarity). D) The same items as in C are sorted according to their similarity to the centroid of the studied items in the language space (i.e., linguistic similarity). The bottom panels report heatmaps illustrating the similarity for each item across all lists: E) the level of visual similarity for the items sorted in each list by visual similarity; F) the level of visual similarity for the items sorted in each list by linguistic similarity; G) the level of linguistic similarity for the items sorted in each list by visual similarity; H) the level of linguistic similarity for the items sorted in each list by linguistic similarity.

To create DRM lists starting from these items, we used an ad-hoc version of the False Memory Generator software (Petilli, Marelli, et al., 2024) an automated tool that analyses similarity relationships between the item representations in a vector space and organises them into DRM lists. As input to FMG, we gave the vector space containing the vision-based vector prototypes (see the section “Vision-based estimate of similarity”) associated with the 1687 selected items. FMG partitioned the space into 12 clusters according to their similarity (Figure 2A). For each of these clusters, FMG selected 50 items close to the cluster centroid (i.e., close space) and 50 random items from the remaining portion of the space (i.e., far space). This resulted in 12 initial lists, each containing 100 items, half derived from the close space (thus similar to each other) and half derived from the far space (thus less similar to the close space items). For each of these lists, FMG selected a subset of items, categorising them into *Studied Stimuli* (i.e., items to be memorised in the encoding phases) and *New Stimuli* (i.e., non-studied stimuli that are presented in the recognition phase only). The Studied Stimuli for each list comprised 30 items, all randomly selected from the close space of their respective DRM list. Among them, a subset of 10 items were selected as *Target Stimuli*, i.e., Studied Stimuli also presented in the recognition phase of the DRM. In contrast, the New Stimuli for each list consisted of the 10 (not selected yet) items closest to the centroid of the *Studied Stimuli* (Děchtěrenko et al., 2021) and 10 random items taken from the far space, thus ensuring that the New Stimuli in each list varied in their degree of similarity to the Studied Items. At the end of this procedure, FMG returned 12 DRM lists of items, each containing 30 items for the encoding phase and 30 for the recognition phase (for a schematic representation of the procedure, see Figure 2B).

Each New Item was associated with two distinct metrics of similarity in relation to the Studied Items within its list. The first metric, vision-based similarity, was computed within the space containing the vision-based prototype vectors for the same items (for details, see the previous section, “Vision-based estimates of similarity”) (Figure 2C). The second metric, language-based similarity, was computed within the space containing the language-based vectors for the selected Italian labels (for details, see the previous section, “Language-based estimates of similarity”) (Figure 2D). For each vector-space, these estimates were computed as the cosine similarity between the vector representation associated with each New Item and the one associated with the centroid (i.e., average vector) of the Studied Items (see Děchtěrenko et al., 2021).

Although visual and linguistic similarities are extracted from different experiential sources and through highly different models, the pattern of the two similarity metrics partially overlaps. As can be seen from the comparisons in Figures 2E-2F and 2G-2H, higher similarity values (blue cells) are consistently more spread on the left parts of the graphs and lower values (yellow cells) on the right part. Within our set of 360 new items, the rank correlation between these similarities indicates a moderate relationship (ρ = .58, *p* < .001). This replicates previous findings in the literature (for a comparison, Günther, 2023 found a rank correlation of ρ = .57 between similarities within a set of 3,000 English word pairs). This outcome is indeed to be expected because language is not at all independent from the physical world we live in, but instead is often used to communicate about this very world (Louwerse 2011). This leads to statistical redundancies between the structure of the physical, directly perceivable world on the one hand, and the structure of language on the other hand (Johns & Jones, 2012; Louwerse, 2011; Rinaldi & Marelli, 2020).

**Figure 3:**
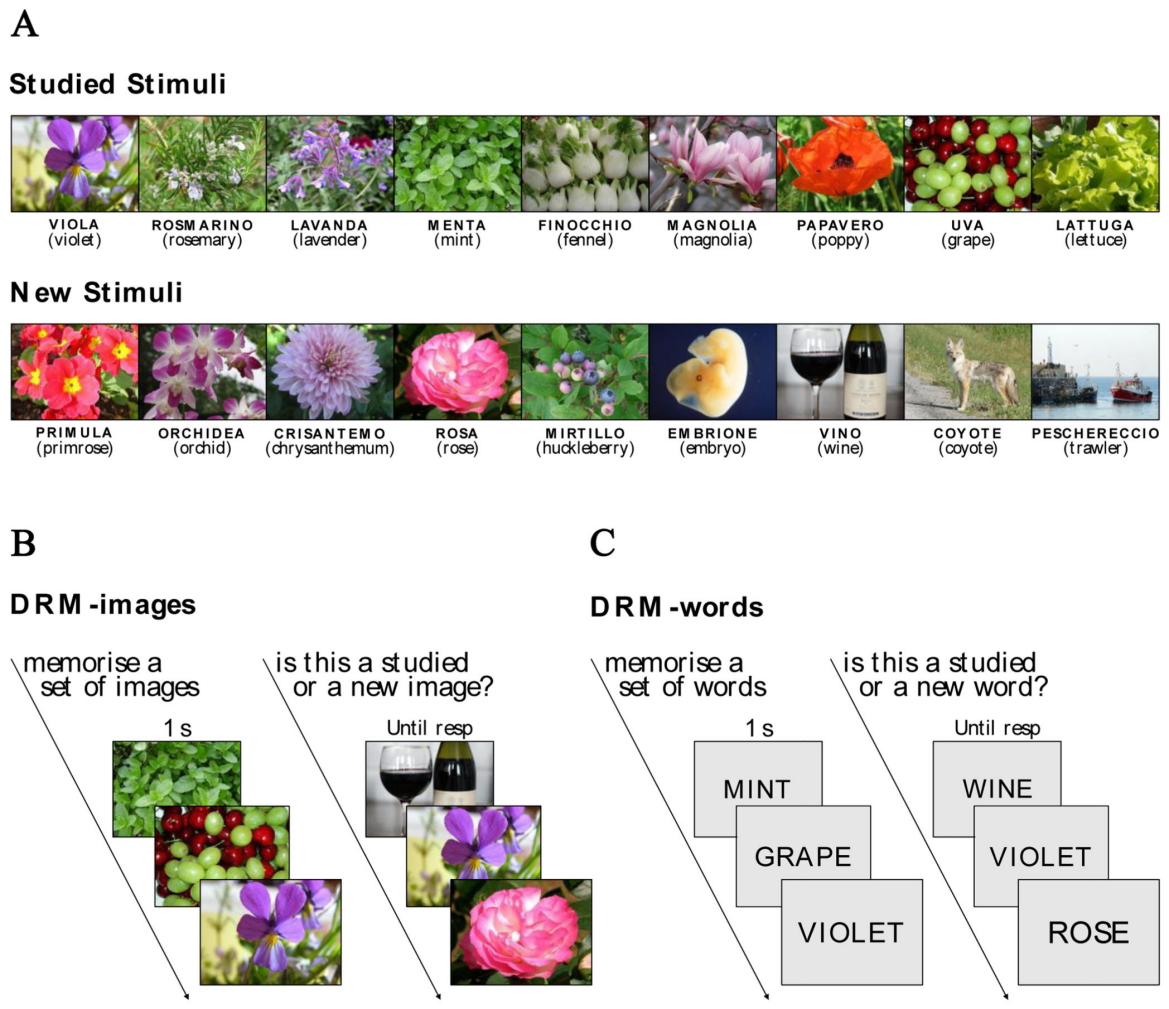
A) Examples of studied stimuli (on the top) and new stimuli (on the bottom) from one of the lists used in the experiments. Each image used in the DRM-image variant (Experiment 1) is accompanied by the corresponding Italian linguistic label used in the DRM-word variant (Experiment 2). The English translation of the word is reported in parentheses. B) Schematic representation of the procedure for Experiment 1. C) Schematic representation of the procedure for the Experiment 2 (the words have been translated here into English for easier understanding but were presented in Italian to participants).

The 12 DRM item lists naturally led to two parallel DRM variants. One variant employed as stimuli the representative images of each word label (Figure 3A-B), while the other used the word labels themselves (Figure 3A-C). These two versions were then used in two independent behavioural experiments: the visual variant in Experiment 1 and the linguistic variant in Experiment 2.

Notably, the DRM list constructed here differs in structure from traditional ones, which commonly include a single critical lure centred around the studied items. Recent studies have already demonstrated the validity of this type of lists, showing them to induce similarity effects in the DRM task comparable to those produced by DRM lists adopting a conventional structure (Děchtěrenko et al., 2021; Rodio et al., 2024; Petilli et al., 2024). Besides its validity, such a structure offers notable advantages, both from a methodological standpoint – as it allows the evaluation of similarity effects in continuous terms – and from a practical perspective – as it enables the flexible construction of DRM lists starting from vector spaces encoding, virtually, any type of object properties (as here, vision-based and language-based semantic properties of objects).

### Experiment 1

In experiment 1, we presented participants with a visual variant of the DRM task. Prior research (Děchtěrenko et al., 2021; Rodio et al., 2024) has shown that, in such visual tasks, the visual similarity between new and studied images predicts false memories: the higher the visual similarity, the higher the probability of falsely recognising a new image as part of the studied list. Experiment 1 was purposefully designed to make potential effects of linguistic similarity to emerge in this purely visual DRM task. To this end, the DRM task of this experiment involved the presentation of images belonging to distinct ImageNet categories (i.e., each image was associated with a distinct word label identifying its content). Subsequently, we examined the impact of language-based similarities on false memories for pictorial stimuli, thus, even though linguistic material was never actually presented to participants. To this aim, we independently quantified vision- and language-based similarities for the labels associated with each image and tested whether the language-based similarity between studied and new images predicted false memories over and above what is predicted by their vision-based similarity.

## Methods

### Participants

40 Italian speakers participated in the experiment (31 identified as women, 8 as men, and 1 as non-binary; age 24.9 ± 6.5 years; education range: high school degree - PhD degree). All demographic information, including gender identity, age, and education level, was collected via free-response items. Participants were recruited through the Sona System platform of the University of Milano-Bicocca (Italy) in exchange for course credits and through online posts on social networks. The study was approved by the ethical committee of the Department of Psychology of the University of Milano-Bicocca (Prot. N. RM-2023-656) and was run in accordance with the principles of the Declaration of Helsinki.

In the present study, statistical analyses were conducted using item-level analysis with Generalised Linear Mixed Models (GLMM). The initial sample size was determined based on Brysbaert and Stevens (2018) recommendations of at least 1,600 observations per condition in item-level analyses using linear mixed models. Starting from this, additional data were collected to minimise the risk of statistical power issues when shifting from Linear Mixed Models to GLMMs. For linear models, the number of parameters to be estimated for continuous predictors is equivalent to the number of parameters to be estimated for two-level factors (for which 3,200 observations are recommended). Given that in our study the effect of interest involves two continuous predictors, following Brysbaert and Stevens’ recommendation (2018), a total of 6,400 observations would be recommended. Accordingly, we recruited 40 participants, each contributing with 240 observations, resulting in 9,600 observations overall. This number substantially exceeds Brysbaert and Stevens’s (2018) recommendation, making the risk of having low statistical power unlikely.

### Procedure and Stimuli

Participants were tested online using Psychopy (v2021.2.3; Peirce et al., 2019) through Pavlovia (https://pavlovia.org/).

The experiment consisted of a practice DRM block preceding twelve experimental DRM blocks. Each block comprised an encoding and a recognition phase separated by a distracting “go-no-go” task.

During the encoding phase, participants were instructed to memorise the images presented on the screen. It is important to emphasise that the DRM task was exclusively visual, as the participants were not exposed to any kind of linguistic material. Each block started with a countdown, signalling the beginning of the encoding phase. The images were presented one at a time in a randomised order at the centre of the screen at 500 x 375 pixels, each for 1000 ms. Each image was preceded by a central fixation cross lasting 500 ms.

The set of images in the recognition phase was presented with the same procedure used in the encoding phase, except that the images remained on the screen until the participants’ response. Here, participants were asked to press “z” if the image was part of the studied list and “n” if the image was new (see Figure 3B for a schematic representation of the procedure used in the experiment).

As typical in the DRM literature, a distracting “go-no-go” task was administered to reduce possible “recency effect” (Capitani et al., 1992) and to monitor participants’ compliance with the experimental procedure. In this task, numbers from zero to nine were randomly presented on the screen, one at a time. Participants had to press “d” only when the number was odd.

### Data Analyses

One participant exhibited low compliance during the distracting “go-no-go” task, as indicated by an accuracy rate lower than 60% and was excluded from the subsequent analyses. All remaining participants showed accuracy in the distracting “go-no-go” task well above the chance level (> 77%), suggesting an adequate level of compliance with the experiment.

Analyses were performed via GLMMs using the lme4 R package (Bates et al., 2014). All GLMMs were run using participants’ responses to the new stimuli in the recognition phases of the DRM as the dependent variable (i.e., ‘new’ responses were scored as 0, while ‘old’ responses were scored as 1). Predictors were standardised to allow comparison of effect sizes. Intercept for participants and list were included as random factors.

As predictors, we used language-based and vision-based similarity estimates between each new item and the studied items within each list (as detailed in section “Computational Framework”). The two predictors were hierarchically included in the model to test whether language-based similarities accounted for variance in the rating values over and above the variance already explained by the vision-based similarities. Indeed, to test the effect of language-based similarity, we first run a baseline model including vision-based similarity as a predictor. We then added the language-based similarity estimates to this model and compared the two models (the baseline and the test model) by estimating their Akaike Information Criterion (which returns an estimate of the quality of the model, the lower, the better, AIC; Akaike, 1987). A ΔAIC = 2 is generally considered as indicative of evidence in favour of the one with the lower AIC (Hilbe, 2011) as it would indicate that the model with the lower AIC is 2.7 times more likely to be a better model in terms of Kullback-Leibler distance from the “real” distribution than the model with the higher AIC (Wagenmakers & Farrell, 2004).

In addition, we also tested for an interaction between vision- and language-based similarity to potentially account for superadditive effects on false memory formation (see, for example, Watson, Balota, & Roediger, 2003, where interactive effects between different types of verbal information can substantially amplify false memories in DRM paradigms). However, including this interactive term did not improve model fit in the experiment and was therefore not included in the final analyses.

Due to the correlation between the two predictors (see section *DRM lists construction*), Variance Inflation Factor (VIF) values were calculated to assess potential issues of multicollinearity.

This study was not preregistered. Supplementary materials related to this article, including the stimuli, generated vector spaces, datasets, and analysis scripts, are openly available on the Open Science Framework (https://doi.org/10.17605/OSF.IO/KU8XV).

## Results

Table 1 presents descriptive statistics for memory performance across 5 categories. Across all categories, the proportion of false recognitions (*pFA*) was significantly higher than zero (all *p*s < .001) with notable variability between categories. Overall, the artefact category exhibited the lowest false recognition rates, paired with consistently high sensitivity. In contrast, the lowest memory performances – both in false recognition and overall sensitivity – were observed in the *plant - plant part* category, for both DRM-images and DRM-words, replicating previous findings showing low memorability for nature scenes (Rust, & Mehrpour, 2020) and plant images (Kramer, 2023).

The GLMM baseline model showed that the effect of vision-based similarity between each new image and the studied images was significant (*b* = 2.242, *z* = 22.813, *p* < .001). This indicates that false recognitions of image stimuli increase with the increasing visual similarity between each new image and the studied images, consistent with previous evidence reported by Děchtěrenko et al. (2021). The comparisons between the baseline model and the test model indicated that language-based similarity significantly improved the baseline model fit (*χ2* (1) = 9.39, *p* = .002). The test model exhibited a lower AIC (4585) compared to the baseline model (4592), with ΔAIC = 7, and Akaike weight of *w* = .976 that indicates that the test model is approximately 40 times more likely to be the best model in terms of Kullback–Leibler discrepancy than the baseline model (Wagenmakers & Farrell, 2004).

The VIF value for the two predictors included in the test model was 1.405, which is well below the accepted threshold of 5 (Menard, 2001), indicating no collinearity issues and supporting the interpretability of the model coefficients.

**Figure 4:**
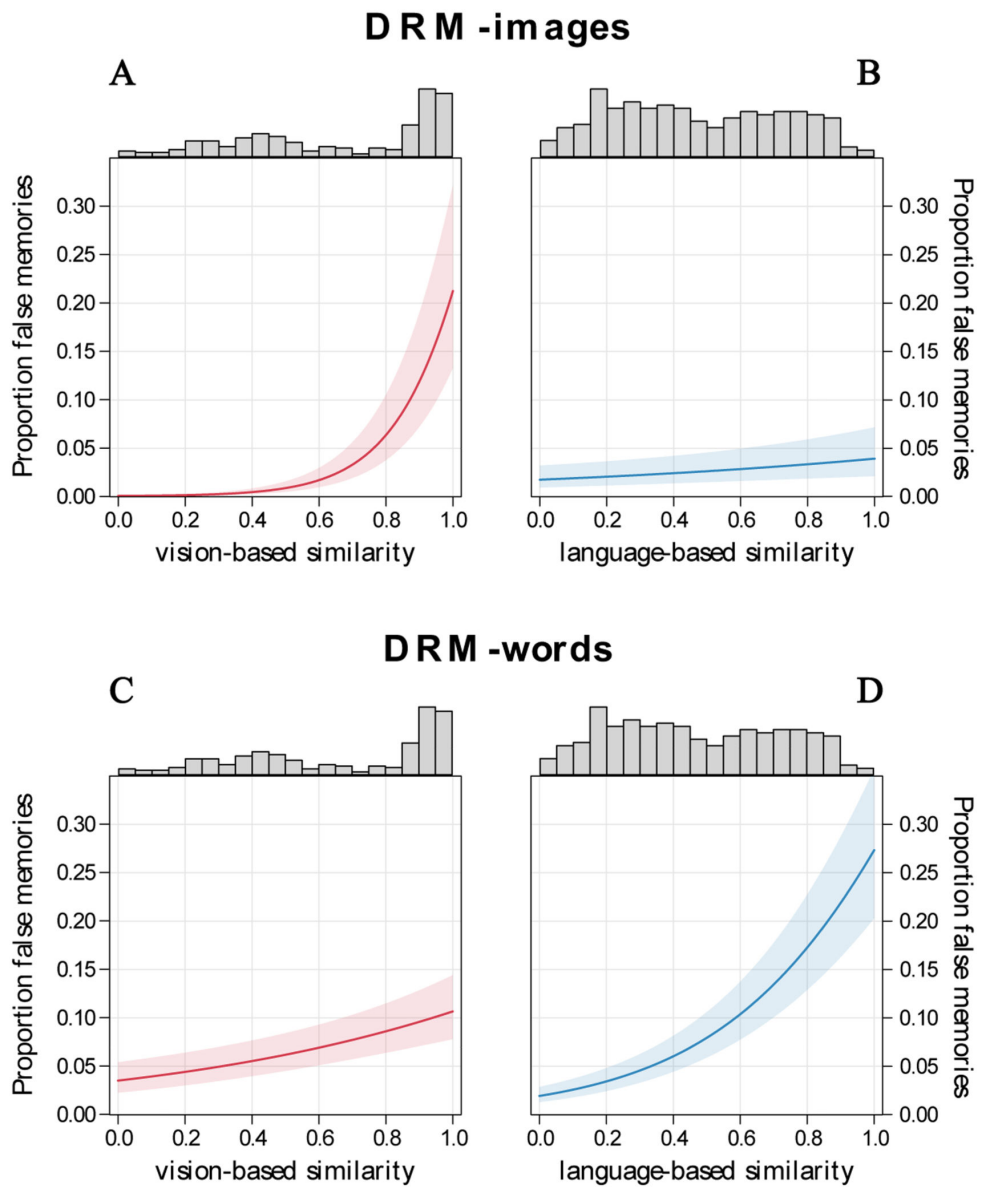
The significant effects of both vision-based (A) and language-based similarity (B) as predictors of the proportion of false recognitions in Experiment 1 (DRM-images). Significant effects of both vision-based (C) and language-based similarity (D) were also found in Experiment 2 (DRM-words). Higher values indicate that the lure is more visually or linguistically similar to (the centroid of the) studied items, whereas lower values indicate less similarity – as defined by distances in the corresponding vector space. Note that cosine similarity estimates extracted from different vector spaces are not directly comparable in absolute terms as they depend on the specific geometry of a space and the orientation of vectors within it. For ease of interpretation, vision-based and language-based cosine similarity values in the figure have been rescaled within a comparable range from 0 to 1 – with 0 representing the minimum level and 1 the maximum level of similarity between lures and the centroid of the studied items observed in our dataset. Thus, a value of 1 does not represent identity, as no vector of lure items is identical to the centroid vector; it simply denotes the highest degree of similarity observed in our data. The distribution of similarity values is shown at the top of each panel.

In the test model, the effects of vision-based and language-based similarity between each new image and the studied images were both significant, although the former had a substantially larger effect (language: *b* = 0.210, *z* = 3.074, *OR* = 1.23, 95% *CI* = [1.08, 1.41], *p* = 0.002; vision: *b* = 2.046, z = 17.577, *OR* = 7.73, 95% *CI* = [6.18, 9.79], *p* < 0.001) (Figure 4 A-B). These results indicate that, besides vision-based effects, false recognition of image stimuli increases with increasing linguistic similarity between the unpresented words corresponding to the presented image stimuli. This supports the multifaceted nature of memory traces, with a reliance on both visual and linguistic prior knowledge in the formation of false memories.

### Experiment 2

Experiment 1 demonstrated that both the vision-based and language-based similarities between new and studied images influence the recognition of image stimuli. This indicates that memorization of visual stimuli involves activation of both visual and language representations associated with the object represented in the image, with this activation exerting a significant influence on false memory formation. This might suggest that the language system plays an overarching role in memory processes, broadly supporting them even when the input is in a different format. However, this does not tell us whether the linguistic system is unique in this capacity or whether the same is true for the visual system. This issue was evaluated in Experiment 2. Here, we administered exactly the same experimental procedure as Experiment 1 with a single exception: the images presented in Experiment 1 were replaced by their corresponding word labels in Experiment 2. The same estimates of vision- and language-based similarities for the presented labels were used to test whether the vision-based similarity between studied and new word stimuli predicted false memories over and above what is predicted by their language-based similarity.

## Methods

### Participants

40 new Italian speakers took part in the experiment (34 identified as women, 6 as men; age 23.05 ± 5.16 years; education range: high school degree - PhD degree), none of whom had participated in Experiment 1. The sample size and the recruitment modality are the same as in Experiment 1.

### Procedure and Stimuli

This experiment was identical to Experiment 1, except for the stimuli used: in Experiment 1, we presented as stimuli images representative of a given category, while in Experiment 2, we presented their corresponding word label (see Figure 3C for a schematic representation of the procedure used in the experiment). The word stimuli were presented at the centre of the screen in uppercase letters using the Open Sans font with a height equal to 10% of the screen height.

### Analyses

One participant exhibited low compliance during the distracting “go-no-go” task, as indicated by an accuracy rate lower than 60% and was excluded from the subsequent analyses. All remaining participants showed accuracy in the distracting “go-no-go” task well above the chance level (> 79%), suggesting an adequate level of compliance with the experiment. In this experiment, we conducted the same analyses as in Experiment 1, with the only difference being the sequence of predictor inclusion in the hierarchical model. Indeed, to test the effect of vision-based similarity, we first ran a baseline model including language-based similarity as a predictor; then, in a test model, we added vision-based similarity and compared the two models in terms of Akaike Information Criterion as described above. We also tested the interaction between vision- and language-based similarity, but as it did not improve model fit, it was not included in the final analyses.

## Results

The baseline model showed that the effect of language-based similarity between each new word and the centroid of the studied words was significant (*b* = 0.992, *z* = 24.626, *p* < .001). This indicates that false recognitions of word stimuli increase with increasing language-based similarity between new words and the studied words, consistent with previous evidence reported by Gatti et al. (2022). The comparisons between the baseline model and the test model indicated that vision-based similarity significantly improved the baseline model fit (*χ2* (1) = 26.6, *p* < .001). The test model displayed a lower AIC (5831) compared to the ‘ aseline’ model (5855), with ΔAIC = 24, and Akaike weight of *w* = .999 indicating that the test model is 216,757 times more likely to be the best model in terms of Kullback–Leibler discrepancy than the baseline model (Wagenmakers & Farrell, 2004) indicating that the test model is 216,757 times more likely to be a better model than the baseline model.

The VIF value for the two predictors included in the test model was 2.218, which is well below the accepted threshold (i.e., < 5; Menard, 2001), indicating no collinearity issues and supporting the interpretability of the model coefficients.

In the test model, the effects of language-based and vision-based similarity between each new word and the studied words were both significant, although the effect of the former was larger (language: *b* = 0.748, *z* = 12.244, *OR* = 2.11, 95% *CI* = [1.87, 2.39], *p* < 0.001; vision: *b* = 0.350, *z* = 5.117, *OR* = 1.42, 95% *CI* = [1.24, 1.63], *p* < 0.001) (Figure 4 C-D). Besides language-based effects, false recognition of word stimuli increases with increasing vision-based similarity between (the referents of) new and studied words. Hence, these results confirm and extend the pattern observed in Experiment 1, unveiling both modality-independent and modality-dependent effects in the formation of false memories.

## Discussion

In our study, we examined how visual and linguistic prior knowledge simultaneously contribute to the formation of false memories. Using data-driven computational models trained on large-scale datasets of words and images, we quantified visual and linguistic similarities to select modality-dependent stimuli (i.e., images and words). These stimuli were then used in two variants of the DRM paradigm, a typical experimental task to induce false memories. Our findings revealed that false memories increase for new words and images that are similar to those in the memorised lists, both visually and linguistically. These findings directly unveil the joint role of multimodal prior knowledge (or schemas) built from linguistic and visual experiences in memory trace formation. Notably, beyond this notation-independent contribution, we also found evidence for modality-dependent reliance. Indeed, the effect of the visual prior knowledge was larger in the DRM task with images, while the effect of linguistic prior knowledge was larger in the DRM with words. Together, these results indicate that memory formation relies on a dynamic integration of prior multimodal information, which becomes flexibly adapted according to the modality-content of the input to be processed at hand.

### The multifaceted nature of memory traces

Our findings support an integrated perspective on the role of prior sensorimotor and language experiences in shaping the structure of human semantic memory. This challenges the extreme views of grounded cognition, which attributes a key role to perceptual simulation (see Barsalou, 1999, 2008; Glenberg, 2015), as well as strong linguistic relativism, which focuses on the contribution of language processing alone (see, Bohnemeyer, 2020; Lucy, 1997; Wolff & Holmes, 2011). Instead, our results indicate that both systems are actively involved in organising our memories. Accordingly, if grounded cognition assumptions were entirely accurate, the effects of vision-based similarity would overshadow language-based similarity across both experiments. Conversely, if linguistic relativity assumptions were entirely accurate, the pattern would be reversed. However, our results clearly showed that both sources of experiential prior knowledge are simultaneously engaged, indicating that neither perceptual simulation nor linguistic processing alone can fully account for the content of memory traces.

This study complements previous evidence pointing to a pervasive interaction between language and perception in giving rise to concept representations (Bedny & Caramazza, 2011; Binder & Desai, 2011; Chatterjee, 2010; Davis & Yee, 2021; De Deyne et al., 2021; Kumar, 2021; Lupyan et al., 2020; Meteyard et al., 2012; Vulchanova et al., 2019; Paivio, 1971). Such interplay between vision and language at the semantic level explains why perceptual effects frequently emerge even in linguistic tasks (e.g., Glenberg & Kaschak, 2002; Lachmair et al., 2011; Petilli et al., 2021) and, at the same time, why language is observed to influence performance in purely perceptual tasks (e.g., Lupyan et al., 2020; Lupyan & Ward, 2013; Samaha et al., 2018; Winawer et al., 2007). These findings, illustrating an interplay between perception and language, are coherently reconciled in the present work, which not only highlights the simultaneous presence of both effects but does so within a unified framework.

In this regard, the most straightforward evidence came from recent works employing explicit similarity judgments (Günther et al., 2023; see also De Deyne et al., 2021). In these tasks, participants typically produce ratings of similarity between pairs of stimuli (i.e., the similarity between the referents of word pairs or the objects presented in image pairs). Here, the contribution of visual and linguistic processes can be inferred by the extent to which similarity estimates based on vision and language uniquely contribute to human ratings. In line with the results reported here, vision- and language-based estimates of similarity were found to independently predict unique variance in human ratings of similarity (Günther et al., 2023). This was observed irrespective of the stimulus format – images or words – and the type of similarity being judged, whether visual or semantic. This aligns with our findings by indicating that similarity judgments invoke hybrid mental representations of the stimuli, built upon both visual and linguistic knowledge, regardless of the type of input stimuli. Such a hybrid representation would also be reflected at the neural level, with fMRI activity patterns elicited by participants reading object names incorporating both visual and text-based representations of the objects (Anderson et al., 2015, 2017).

Notably, our study integrates well-established principles from two distinct theoretical traditions that have rarely converged in the literature: false memories and conceptual representations. As mentioned above, the idea that semantic memory operates through the reactivation of multimodal traces is consistent with existing literature on conceptual representations (e.g., Paivio,1971). Here, we further highlight how such reactivation (1) extends to the formation of false memories following well-established principles of similarity underlying false memory occurrence and (2) is specifically modulated by the stimulus format. Perhaps more crucially, the activation of a hybrid-like memory trace is observed in a task that does not explicitly require any similarity judgement, but rather to simply memorise items without explicit requirements on semantics.

### Flexible contribution of visual and linguistic prior knowledge

Another relevant aspect highlighted by our study is the independent and flexible contribution of visual and linguistic systems during the memory task. Indeed, the weight of visual and linguistic effects was adaptively calibrated according to the demands of the task at hand. This highlights a partial dissociation in how the two systems contribute to the formation of memory traces as a function of the specific task requirements. This also aligns with evidence from explicit similarity judgement tasks. Accordingly, vision-based estimates of similarity are more accurate in predicting human ratings of visual similarity compared to semantic similarity (Günther et al., 2023). Conversely, language-based estimates of similarity are more effective when participants judge pairs of stimuli based on their semantic rather than visual similarity (Günther et al., 2023). Notably, even though here participants performed exactly the same memory task, the format of the presented stimuli – image or text – was critical in determining the relative influence of visual and linguistic prior knowledge.

The flexible activation of the vision and language systems in relation to the format of stimuli is not limited to tasks involving explicit responses but is also traceable at an implicit level, as evidenced by results from the priming paradigm (e.g., Tulving & Schacter, 1990). In a standard priming paradigm, a ‘prime’ stimulus is presented before a ‘target’ stimulus to which participants must respond. The type of response depends on the specific requirements of the task (Carr et al., 1982; Hutchison et al., 2013; Meyer & Schvaneveldt, 1971; Sperber et al., 1979). For example, participants may be asked to name the target or to decide whether it is a real *vis-à-vis* non-real stimulus (e.g., a scrambled image or a string of letters that doesn’t form a real word). In this task, typically, responses to the target are facilitated, the more similar the prime and the target are. This is commonly explained by the prime pre-activating mental representations related to it, thus facilitating access to targets similar to the prime (Hutchison et al., 2013). Interestingly, when the prime and target stimuli were presented as images, only vision-based similarity was found to predict the priming effect (Günther et al., 2023). Conversely, when the stimuli were words, language-based similarity effects were more pronounced and vision-based similarity effects tended not to emerge (Pecher et al., 1998) or appeared subtler and selectively under specific task variants (i.e., in lexical decision but not in word naming) and timing (i.e., when stimulus onset asynchrony is short, but not long) conditions (Petilli et al., 2021). Here, similarly to these previous findings, the visual and linguistic traces are adaptively activated depending on the format of the input stimulus. However, contrary to these previous findings, both effects related to visual and linguistic prior experience consistently emerged, likely because of the more controlled experimental design and the non-reliance on chronometric methods.

It is also worth considering that a pronounced asymmetry in the contribution of visual and linguistic prior knowledge emerged across the two experiments. In the image-based task (Experiment 1), visual similarity was by far the stronger predictor compared to linguistic similarity, whereas in the word-based task (Experiment 2), the contributions of visual and linguistic similarity were much more balanced. Such a result may suggest that in a purely perceptual task, same-modality representations play a prominent role, whereas a linguistic task might engage more multimodal representations (that are thus less constrained by the input modality). This pattern is particularly interesting as it adds specific details regarding how the degree of access or prioritisation of memory traces from different domains varies with stimulus modality. Nevertheless, this asymmetry should be interpreted with caution, as it may alternatively be due to the DRM lists being constructed from a visual vector space (please see the section “DRM lists construction” for details on the procedure employed to select stimuli for DRM lists in our study), which could make the experiments more sensitive to visual effects than to linguistic ones. Further studies are therefore needed to explore this asymmetry.

Note that our study benefits from a large number of observations collected across participants, which provides high statistical power. This is particularly advantageous because it allows for the detection of even small effects (Chen, Cohen, & Chen, 2010), as in the case of the language similarity effect in the image-based DRM task. Remarkably, despite these experiments being optimised for detecting visual rather than linguistic effects, language-based similarity effects emerged even when the input stimuli were presented as images and still outperformed the visual effects when the input stimuli were in the form of words. This indicates that while we cannot exclude the possibility that the chosen procedure may have led to potentially underestimating the impact of language-based similarity effects, such a bias did not prevent significant linguistic effects from emerging.

### Prior knowledge captured by computational models

Our study employed advanced computational techniques to investigate memory, assessing for the first time the joint contribution of visual and linguistic prior knowledge to the occurrence of false memories. This approach allows to quantify the similarity between studied and new items on a continuous scale using independent data sources. In contrast to human ratings, this method employs data from text corpora and image databases, representing proxies for the human linguistic and visual prior experience. Previous research using distributional semantic models has indeed shown that prior linguistic similarity between words can predict false memories in word-based DRM memory tasks (Gatti et al., 2022). In a similar vein, using Convolutional Neural Networks (CNNs), it has been demonstrated that visual similarity can predict false memories in image-based DRM tasks (Děchtěrenko et al., 2021; see also Rodio et al., 2024). Our approach builds upon these earlier works and extends prior findings by integrating visual and linguistic knowledge within the same – image-based and word-based – memory paradigms. Crucially, this integration enabled a direct comparison of their relative effects both within and across their respective stimulus formats, making it possible to evaluate multimodal effects across different stimulus modalities. Indeed, through this approach, it is therefore possible to approximate mental representations for image objects and word referents in a parallel manner, enabling us to evaluate the involvement of vision and language systems in shaping the same representations in different task conditions.

In addition, it is important to highlight that using a data-driven and objective approach to establish similarity between representations of objects – rather than human intuitions – is highly desirable for psychological studies, as it allows us to bypass the loophole of predicting behavioural data (here false memories) from other behavioural data (e.g., human ratings; for a discussion see, Jones, Hills, et al., 2015; Westbury, 2016). This method, therefore, protects against the concerns of objectivity and reliability raised by adopting human ratings (Simmons & Estes, 2008; Tversky, 1977).

Furthermore, it is worth mentioning that the current stimulus selection procedure (based on Petilli et al., 2024) has led to the adoption of a rich and diverse set of item lists. This diversity represents a meaningful step forward in achieving a more ecologically valid sample of stimuli, especially when compared to the typical approaches adopted in previous research, which often over-rely on widely used association norms and the stimulus lists derived from them. While these established norms remain highly relevant and are still widely adopted in the field, their creation and use are inevitably subject to temporal, geographical, and practical constraints.

### Possible directions for future studies

Notably, this study opens several avenues for future research. For example, it would be relevant to assess whether flexibility in the contribution of these systems is also observed in response to more subtle changes in experimental procedures without necessarily altering the format of the stimuli. Indeed, such an experimental setup is perfectly suited to evaluate the flexible contribution of visual and linguistic systems as a function of other task variants or manipulations, while keeping the stimulus format unchanged. For example, McDermott and Roediger (1994) examined how instructions influence the activation of visual and linguistic traces during priming tasks. They demonstrated that imagination instructions can increase cross-modal priming effects (e.g., words priming picture fragments or pictures priming word fragments) compared to conditions where such instructions are not provided (Weldon & Roediger, 1987). A similar approach could be applied here by using lists of words (or images) presented more slowly (e.g., at a rate slower than one per second) alongside instructions to form mental images of the referents of those words (or the word labels of those images). This experimental setup would allow for an evaluation of whether such a manipulation leads to stronger cross-modal effects compared to a condition where stimuli are presented without imagery instructions, thus providing insights into how instruction manipulations influence the balance between the contribution of visual and linguistic systems in memory formation.

Importantly, – using Marr’s terminology about the levels of analysis of information-processing systems (1982) – these findings advance knowledge at the *computational* level by demonstrating the existence of the phenomenon and providing predictions based on the model’s results, without addressing the *algorithmic* level, which explains how exactly these processes are implemented. Other lines of research could leverage our approach to take a step further into the investigation of the experiential mechanisms that lead to activation of multimodal representations in the DRM task. For example, one plausible hypothesis is that repeated exposure to visual stimuli paired with corresponding linguistic labels (Zwaan & Madden, 2005) may play a role in strengthening the connection between visual and linguistic counterparts and, in turn, facilitating the spreading of cross-modal activations in the DRM task. Alternatively, such an effect might arise from modality-specific expertise (e.g., the familiarity we have with the linguistic label or with the visual referents of the concept), rather than from cross-modal connections formed by pairing visual stimuli and linguistic labels in direct experience. In this case, having higher familiarity of a concept in one of the two modalities might lead to a greater reliance on the corresponding memory system when processing the underlying concept representation. Note, however, that while having prior direct experience with the words or their visual referents might plausibly play a role, it may not be a necessary requirement for spreading activation between modalities. Previous research has shown that we can capture and learn statistical relationships between the way we use language and the perceptual experience we have of the world. These learned associations can be productively applied to activate visual information, even for word referents for which we have no direct experience (e.g., abstract concepts; Johns & Jones, 2012; Louwerse, 2011; Günther, Petilli, Vergallito, et al., 2020) or for the referents of words encountered for the first time (e.g., through form-meaning associations – Dingemanse, Blasi, Lupyan, et al., 2015 – or through extraction of semantic information from the lexical or visual contexts in which the stimulus appear; Sloutsky, Yim, Yao et al., 2017; Lazaridou, Marelli, & Baroni, 2017; de Varda, Petilli, & Marelli, 2023). This suggests that cross-modal effects regarding a single item might be hypothesised even in the absence of prior linguistic and/or visual experience with the word label or the visual referent to which the item refers. Building on the findings that have emerged in this study, future research could incorporate measures to preliminarily evaluate the familiarity and strength of associations between the visual and verbal stimuli used in the experiment, thereby enabling an assessment of the role of this pre-existing knowledge about the stimuli in modulating the observed effects of similarity.

Finally, the versatility of such an experimental approach could extend the investigation to the role of experiential prior knowledge derived from other modalities. Indeed, the procedure used here can be flexibly adapted to design lists of stimuli optimised to evaluate the effect of non-visual sensory modalities in triggering the DRM false memory effect. Such an extension would provide a more comprehensive understanding of how sensory systems, alongside language, flexibly contribute to memory processes rather than focusing solely on the dominant modality of vision.

## Conclusions

To conclude, these findings are novel in two main respects. First, they serve as the first demonstration, to our knowledge, of how effectively the phenomenon of false memories can be leveraged to gain insights into the organisation of traces in human memory. Secondly, our study offers compelling evidence that enhances our understanding of how semantic memory is organised through the continuous interplay between visual perception and language. Indeed, they demonstrate that both visual and linguistic relations between newly encountered information and previously assimilated knowledge jointly contribute to memory distortions. This suggests that gaps in our memory are filled with details obtained from both linguistic and visual experiences, indicating that the framework for encoding new information is grounded in both these experiential sources. Importantly, our findings also demonstrate that the format of the newly encountered information significantly influences the extent of the relative involvement of language and vision prior knowledge. This adds a layer of complexity to the interaction between language and vision within semantic memory.

## Figures and Tables

**Table 1:** Descriptive statistics are presented separately for Experiment 1 (DRM-images) and Experiment 2 (DRM-words) based on the distribution of 360 items presented in the recognition phase, grouped across six WordNet categories (animal, artefact, food, person, plant/plant part, and structure; for details, see the section “DRM list construction”). Measures include the mean and standard deviation (SD) of the proportion of hits (pHits), false alarms (pFA), and detection sensitivity (A’) across participants.

	DRM-images			DRM-words		
	*pHit*	*pFA*	*A’*	*pHit*	*pFA*	*A’*
*animal* (N = 66)	.72 (.22)	.17 (.16)	.86 (.08)	.77 (.14)	.17 (.12)	.87 (.08)
*artifact* (N = 117)	.76 (.18)	.05 (.07)	.92 (.05)	.7 (.16)	.08 (.08)	*.89 (.07)*
*food* (N = 26)	.81 (.2)	.1 (.14)	.9 (.08)	.7 (.24)	.08 (.11)	.87 (.12)
*person* (N = 38)	.76 (.18)	.14 (.15)	.88 (.07)	.72 (.16)	.15 (.1)	.86 (.07)
*plant/plant part* (N = 30)	.74 (.23)	.19 (.16)	.85 (.1)	.78 (.17)	.19 (.14)	.86 (.09)
*structure* (N = 50)	.71 (.23)	.12 (.1)	.87 (.08)	.8 (.15)	.13 (.1)	.9 (.07)
*uncategorised* (N = 33)	.72 (.22)	.25 (.17)	.81 (.12)	.68 (.2)	.2 (.14)	.81 (.13)

## Data Availability

Supplementary materials related to this article, including the stimuli, generated vector spaces, datasets, and analysis scripts, are openly available on the Open Science Framework (https://doi.org/10.17605/OSF.IO/KU8XV).

## Public Significance

This study provides the first demonstration of how effectively the phenomenon of false memories can be leveraged to gain insights into the multimodal organisation of human memory. The findings indeed reveal that both visual and linguistic relations between newly encountered information and previously assimilated knowledge jointly contribute to memory distortions. The gaps in our memory are thus filled with details obtained from both prior linguistic and visual experiences, demonstrating, in turn, the multimodal nature of memory traces. Additionally, this study demonstrates that the format of the newly encountered information significantly influences the extent of the relative involvement of prior knowledge, showcasing the flexibility of the human memory system in adapting to specific task demands.

## Footnotes

1 Note that, as part of the preprocessing step in the VGG-F algorithm, all images are scaled to a fixed size of 224 x 224 to standardize the input layer (Chatfield et al., 2014). Therefore, our resizing step does not remove any information that the VGG-F algorithm might use.
